# Dual Radiolabeling as a Technique to Track Nanocarriers: The Case of Gold Nanoparticles

**DOI:** 10.3390/molecules200712863

**Published:** 2015-07-16

**Authors:** Clinton Rambanapasi, Nicola Barnard, Anne Grobler, Hylton Buntting, Molahlehi Sonopo, David Jansen, Anine Jordaan, Hendrik Steyn, Jan Rijn Zeevaart

**Affiliations:** 1 DST/NWU Preclinical Drug Development Platform, Faculty of Health Sciences, Potchefstroom Campus, North-West University, Potchefstroom 2531, South Africa; E-Mails: Nicola.Barnard@nwu.ac.za (N.B.); Anne.Grobler@nwu.ac.za (A.G.); 24861820@nwu.ac.za (H.B.); janrijn.zeevaart@necsa.co.za (J.R.Z.); 2 Radiochemistry Department, South African Nuclear Energy Corporation (SOC) Ltd., P. O. Box 482, Pretoria 0001, South Africa; E-Mails: Molahlehi.Sonopo@necsa.co.za (M.S.); david.r.jansen@gmail.com (D.J.); 3 Laboratory for Electron Microscopy, Chemical Resources Beneficiation Group, Potchefstroom Campus, North-West University, Potchefstroom 2531, South Africa; E-Mail: Anine.Jordaan@nwu.ac.za; 4 Statistical Consultation Services, Potchefstroom Campus, North-West University, Potchefstroom 2531, South Africa; E-Mail: Faans.Steyn@nwu.ac.za

**Keywords:** gold nanoparticles, dual radiolabeling, biodistribution profiles, Sprague Dawley rats

## Abstract

Gold nanoparticles (AuNPs) have shown great potential for use in nanomedicine and nanotechnologies due to their ease of synthesis and functionalization. However, their apparent biocompatibility and biodistribution is still a matter of intense debate due to the lack of clear safety data. To investigate the biodistribution of AuNPs, monodisperse 14-nm dual-radiolabeled [^14^C]citrate-coated [^198^Au]AuNPs were synthesized and their physico-chemical characteristics compared to those of non-radiolabeled AuNPs synthesized by the same method. The dual-radiolabeled AuNPs were administered to rats by oral or intravenous routes. After 24 h, the amounts of Au core and citrate surface coating were quantified using gamma spectroscopy for ^198^Au and liquid scintillation for the ^14^C. The Au core and citrate surface coating had different biodistribution profiles in the organs/tissues analyzed, and no oral absorption was observed. We conclude that the different components of the AuNPs system, in this case the Au core and citrate surface coating, did not remain intact, resulting in the different distribution profiles observed. A better understanding of the biodistribution profiles of other surface attachments or cargo of AuNPs in relation to the Au core is required to successfully use AuNPs as drug delivery vehicles.

## 1. Introduction

The use of engineered nanomaterials, such as gold (Au) nanoparticles (AuNPs), promises to have a great impact on the field of nanomedicine and nanotechnologies. As a result, AuNPs have become an on-going area of research for a wide range of biomedical applications, such as plasmon-based labeling and imaging, diagnostics and therapeutics [1,2,3]. AuNPs’ unique surface, electronic and optical properties, as well as their apparent biocompatibility [4] make them ideal drug delivery vectors [5,6,7,8]. However, their biocompatibility and toxicity have recently been questioned [9,10], and currently, there is no consensus on their biodistribution [4,11,12,13]. This can be attributed to the use of different methodologies with a diversity of objectives that do not collate easily into a single general conclusion. The lack of correlation between *in vitro* and *in vivo* toxicity results further complicates matters.

In biodistribution and toxicity studies, it is necessary to accurately determine the amount of Au in various tissues/organs. The quantification of the other components of an AuNP drug delivery vesicle, the surface coating and surface attachments or the cargo, can assist in the elucidation of potential toxicity mechanisms. Several techniques have been used to measure the content of Au in rodents, for example; inductively-coupled plasma mass spectroscopy (ICP-MS) [10,14,15,16,17,18], atomic absorption spectroscopy (AAS) [19], radioactive analysis (RA) using gamma spectroscopy [20,21,22,23] and instrumental neutron activation analysis (INAA) [24,25]. Gamma spectroscopy and INAA are preferred analytical techniques in biodistribution studies due to the lower limits of detection compared to AAS and ICP-MS. Gamma spectroscopy offers the added advantage of a quick and relatively simple sample preparation. However, all of these quantification methods mentioned lack the ability to track and quantify the other components/surface attachments of AuNPs simultaneously *in vivo*.

Whilst the use of a single radiolabel is common [13,20,23], to the best of our knowledge, there are no published studies using dual radiolabeling to determine the biodistribution profiles of the different components in a multi-component systems for AuNPs. However, dual radiolabeling has been reported before to study the biodistribution of the components of a vaccine system (both adjuvant and antigen) [26] and for AuNPs with two radiolabels for use in single-photon emission computed tomography (SPECT) for bioimaging applications in diagnostics [27]. An approach similar to the one we are taking in this study was done for superparamagnetic iron using ^59^Fe for the nanoparticle core and labeled surface attachments [28,29].

An understanding of the biodistribution profile of each component (Au core and any surface attachments) would be ideal, as this will enable any observed end organ toxicity to be attributed to the whole system or a part thereof. This can be achieved by radiolabeling each of the desired components; in this case, the Au core and the citrate surface coating. The methods used by Hirn *et al.* of radiolabeling AuNPs by irradiation of a pellet of AuNPs (^197^Au (n,γ) ^198^Au) [20,23] cannot be used for dual radiolabeling of the Au core and the surface coating, as neutron activation only produces ^198^Au. In this study, the Au was radiolabeled using ^198^Au, while [^14^C]citrate was used for the citrate surface coating.

The aim of the present study was to synthesize dual-radiolabeled AuNPs and to determine the biodistribution profiles of the Au core and citrate surface coating, while investigating the influence of the route of administration and the dose level. Well-characterized 14-nm AuNPs that were dual-radiolabeled were administered to healthy male Sprague Dawley rats intravenously and orally. The study served as a proof of principle that dual radiolabeling can be used to determine the biodistribution profiles of the different components of a multi-component system. Therefore, in this study, the acute biodistribution profiles of the Au core and citrate surface coating after oral and intravenous (*i.v*.) administrations are presented. Future studies must investigate the biodistribution of Au after multiple doses and assess its biopersistence, while focusing more on toxicity endpoints.

## 2. Results

### 2.1. Synthesis and Characterization of AuNPs

**Figure 1:**
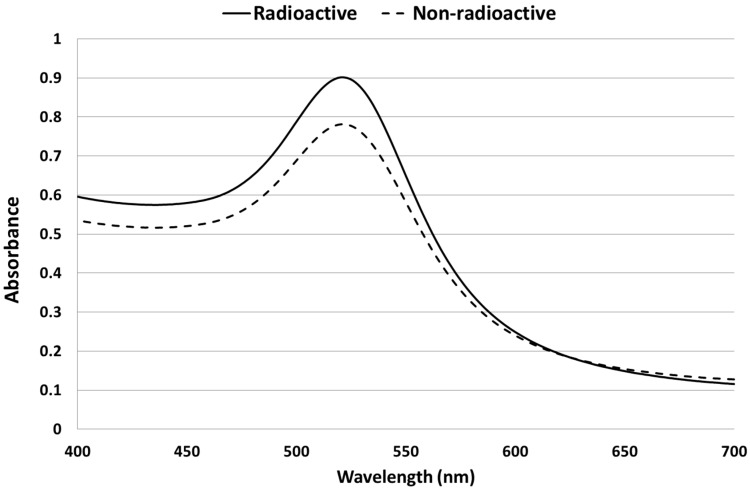
UV-Vis spectra of radioactive (**continuous line**) and non-radioactive (**dashed line**) AuNPs. The measurements were done after the synthesis of both samples.

AuNPs were synthesized from both radioactive and non-radioactive precursors using the citrate reduction method. The UV spectra peaks in Figure 1 were similar for both radioactive and non-radioactive AuNPs.

The UV peak was around 520 nm as expected for this particle size range, whilst the dispersion quality was confirmed by the absence of absorbance at wavelengths greater than 600 nm [30].

**Figure 2:**
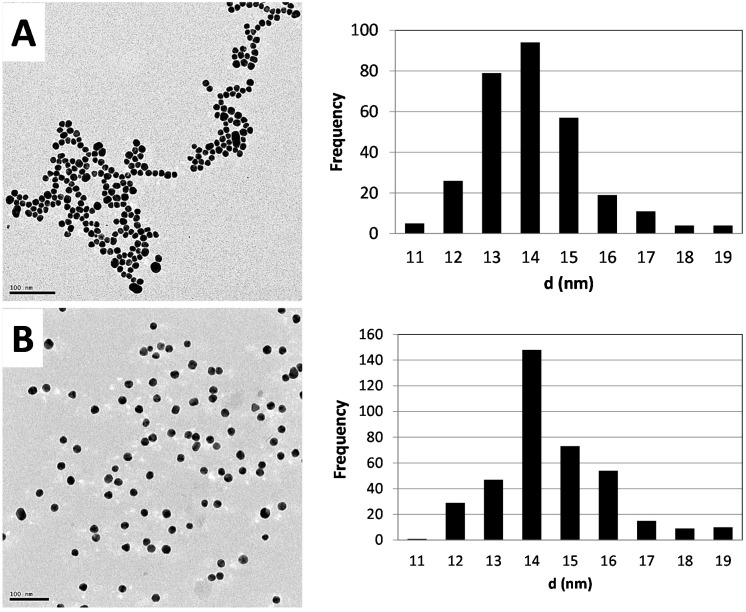
Morphology and size distribution profiles of the synthesized AuNPs fabricated from natural gold (**A**) and radioactive gold (**B**). On the left are TEM images, with the particle size distribution plots on the right.

From the TEM images and the particle size distribution plots (see Figure 2), it can be seen that the morphology and primary particles size distribution of 14 ± 1.2 nm and 14 ± 1.5 nm for radioactive and nonradioactive AuNPs, respectively, are similar/comparable. Hydrodynamic sizes of 25 nm for the non-radioactive preparation and 23 nm for the radioactive samples with zeta potentials of −50.9 mV and −48.9 mV were found, respectively.

The specific activity of the ^198^Au was 108 GBq/g, with an activity concentration of 25.9 MBq/mL and an isotope ratio (^198^Au:^197^Au) of 1.45 × 10^−5^. The specific activity of the ^14^C was 52.43 MBq/g, with an activity concentration of 0.054 MBq/mL and an isotope ratio (^14^C:^12^C) of 0.8.

### 2.2. Biodistribution of Gold vs. Citrate in the Rat

#### 2.2.1. Dosimetry

Table 1 summarizes the main dosimetric features of the [^14^C]citrate-[^198^Au]AuNPs used. The surface area and number of nanoparticles were calculated using the initial mass of Au used in the synthesis.

**Table 1:** Characteristics of the dual-radiolabeled AuNPs used in the study at the 2 dose levels used (high and low). The surface area of the AuNPs was calculated using the primary size determined using TEM.

		Dose		
		High	Low	
Administered radioactivity per rat (MBq)	^198^Au	12.95		1.22
	^14^C	0.027		0.0027
Administered mass per rat (μg)	Au	90		9
	Citrate	520		52
Administered number of AuNPs per rat		3.27 × 10^12^		3.327 × 10^11^
Administered surface area (cm^2^) of AuNPs per rat		20.16		2.02

#### 2.2.2. Biodistribution Profiles

**Figure 3:**
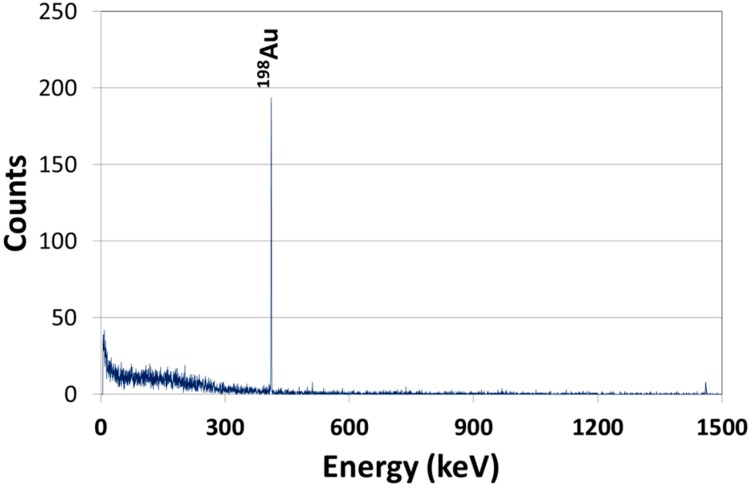
Gamma spectrum of the radioactive gold nanoparticles used in the animal study.

In order to ensure that the signal measured was only from gold and that there were no interferences that may impede the reliability of the results, a gamma spectrum was measured (see Figure 3). The peak at 411 keV is for ^198^Au; the absence of other peaks shows that the gold used in the experiments did not have impurities, and only gold was quantified in the work.

**Figure 4:**
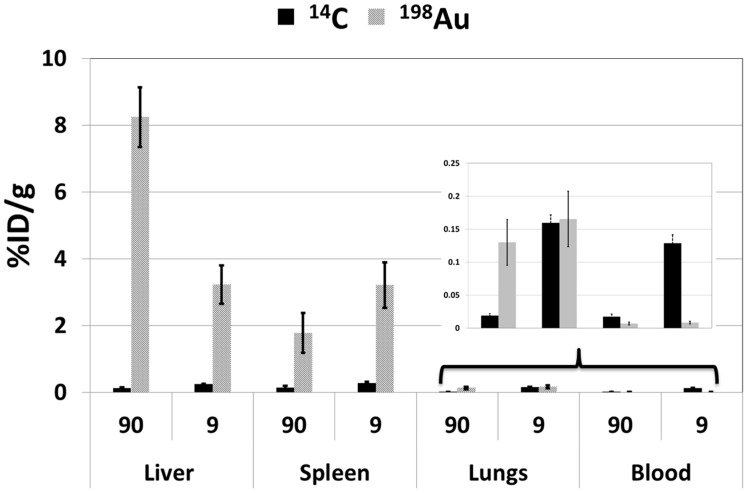
Amounts of gold and citrate expressed as a percentage of the injected dose per gram of organ/tissue (%ID/g) in the liver, spleen, lungs and blood, 24 h after intravenous administration of dual-radiolabeled AuNPs. Results are expressed as the mean ± SD.

The biodistribution profiles of both the nanoparticle core and surface coating were investigated, with the route of administration and dose level as variables. The amount of Au and citrate was determined using γ-spectroscopy for ^198^Au and liquid scintillation for ^14^C at 24 h post a single dose administered intravenously and orally. The amounts of ^198^Au and ^14^C in the liver, spleen, lungs and blood were determined in the analysis. Oral administration of AuNPs resulted in no systemic uptake of ^198^Au; thus, no ^14^C was measured in the oral group. Activities of the ^198^Au were only measured/detected in the stomach and other parts of the GI tract, with no measurable activity in all other organs with less than 0.001% injected dose (ID)/g in the blood, liver, spleen and lungs (results not shown). The inclusion of the oral group was because some measurable systemic uptake was expected, as described in the literature [24]. However, our findings correspond well with the results reported in previous studies and are attributed to the size of the particles used in the study [23,24,31]. Therefore, only results from the two intravenous groups (see Figure 4) are reported here. The results are expressed as the percentage injected dose per gram of organ/tissue (%ID/g) for the Au and citrate. The amounts of ^198^Au measured in the urine and feces were used to perform a mass balance for Au.

##### Liver

The liver had the highest %ID/g of gold when administered in both 90-μg (high) and 9-μg (low) doses, with 8.2% and 3.2%, respectively. The %ID/g of citrate was less than 0.5% irrespective of the administered dose. The difference in the %ID/g of the Au was statistically significant using both the Mann–Whitney and Student *t*-tests; the *p*-values were calculated to be 0.03 and 0.0004, respectively, at the two dose levels used.

##### Spleen

The spleen had the second highest %ID/g of gold. However, contrary to the liver, the %ID/g was inverted relative to the administered doses, with 1.8% for ID of 90 μg and 3.2% when 9 μg were administered. The differences were not statistically significant. The values for the citrate were also determined to be under 0.5%.

##### Lungs

The %ID/g of both the Au and citrate were under 0.25% in the lung tissue. The biodistribution pattern was comparable between the Au and citrate only for the administered dose of 9 μg (low dose). The difference in the %ID/g of the Au was significant using both Mann–Whitney and Student *t*-tests; *p*-values of 0.03 and 0.002, respectively, at the two dose levels used.

##### Blood

The %ID/g of citrate was low (≤0.15%) and that of Au even lower (≤0.02%). There was not a statistically significant difference in the %ID/g of citrate at the two dose levels. The biodistribution profiles of the Au were independent of the dose quantity.

##### Summary of Biodistribution Profiles

**Figure 5:**
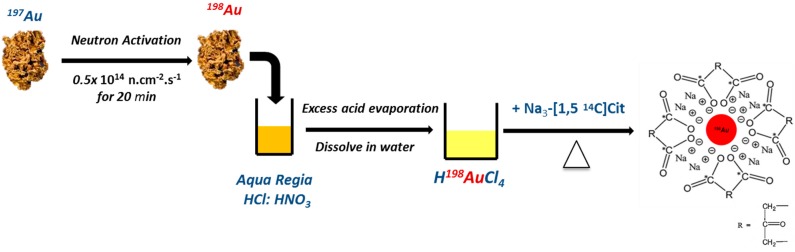
Schematic showing the synthesis of dual-radiolabeled AuNPs starting with the stable ^197^Au isotope of gold. The neutron activation step is unique to the radioactive synthesis.

In general, Au and citrate had unique biodistribution profiles, as shown by the differences in %ID/g portrayed in Figure 5 with the exception of the 9-μg administered dose in the lungs, where the %ID/g values were comparable.

The biodistribution profile of the Au varied based on the dosing level, 90 μg *vs.* 9 μg. The ratios of %ID/g of Au between the 90 μg and 9 μg dose were: liver, 2.6; spleen, 0.6; lungs, 0.8; blood, 0.8; whilst for citrate, the ratios were: liver, 0.5; spleen, 0.5; lungs, 0.1; blood, 0.1.

## 3. Discussion

The need for extensive biodistribution studies to assess the safety of AuNPs can never be over emphasized, as this will ensure that AuNPs reach the clinic faster. With no consensus on the toxicity profile of AuNPs, a need to understand the biodistribution profile of each component of the AuNP system becomes apparent. It has been generally accepted that surface functionalization is an important determinant of the *in vivo* dynamics and toxicity [11,20,32]. In this study, we synthesized AuNPs, both non-radioactive and radioactive, and compared the two formulations to assess the impact of using radioactive precursors on the physico-chemical properties. The dual-radiolabeled AuNPs were used to determine the biodistribution profile of the Au core using ^198^Au and surface coating using [^14^C]citrate. The influences of the dose and route of administration were also investigated.

The use of radioactive precursors had no impact on the quality attributes of the synthesized dual-radiolabeled AuNPs. This was shown when the physico-chemical properties of the non-radioactive and radioactive AuNPs were compared. The UV/Vis spectra of the non-radioactive and radioactive batches were comparable and characteristic of the 14-nm size range, which has a defined plasmon resonance peak maxima around 520 nm [19]. The absence of secondary peaks at wavelengths higher than 600 nm also confirms the absence of agglomerates and/or aggregates in the suspensions [30]. The polydispersity index (PDI), a measure of monodispersity obtained with the Zetasizer Nano ZS, also showed that the suspensions were free of agglomerates/aggregates. The zeta potential was as expected: a high negative charge due to the negative charge of the citrate surface coating. This was comparable for both the non-radioactive and radioactive AuNPs. The molar ratio of the hydrogen chloroauric acid:citrate used in the synthesis of the AuNPs yielded nanoparticles with a core diameter around 14 nm, which is consistent with the sizes obtained by other researchers when similar molar ratios were used [15,16,33].

The radiotracers used in the synthesis of the dual-radiolabeled [^14^C]citrate-[^198^Au]AuNPs were well controlled and were adjusted to meet the varying requirements. The photons emitted during the decay of ^198^Au have energies that can be detected by a gamma camera; thus, a change in the activity concentration of the Au during uptake in the various organs can be imaged. The method used in this work solves some challenges that are normally encountered when other ways of incorporating radiotracers into AuNPs are used. Agglomeration and/or aggregate formation when synthesized AuNPs are irradiated to neutrons activate the Au core [20,21,22,23]. The activity of both labels was homogenously distributed in the solution. This was shown experimentally when the doses were measured using both volume and radioactivity. There was a correlation between the expected and determined value for each dose using ^198^Au.

In this study, the biodistribution profiles observed for the Au core and surface coating were very different. Use of surface attachment as the radiotracer has been done [34] and suffers the disadvantage of misinterpretation of the biodistribution profiles. The radiotracers can be displaced from the core due to the formation of a bio-corona [35,36]. Usually, the biodistribution of the radiotracer is assumed to represent that of the Au core and the whole nanoparticle system. From our results, it is seen that surface attachments will not have to have the same biodistribution profile as that of the core or carrier molecule used to transport it. Caution must therefore be exercised when interpreting the results of biodistribution and toxicity studies of AuNPs with surface attachments that will not be present in those intended for biomedical applications. The addition of different surface attachments will most likely alter the biodistribution and toxicity profile of AuNPs *in vivo*, as surface chemistry plays an integral part in the toxicity and biodistribution of AuNPs and other nanomaterials [37].

The biodistribution profiles of the Au core and citrate surface coating were different in the organs/tissues used in the analysis. This can be explained by the formation of the bio-coronas around the nanoparticle core [38,39,40,41,42,43], which results in the dissociation of the surface attachment from the core. These results indicate that during the synthesis and design of therapeutic agents, the type of interaction between the Au surfaces and “cargo” should be carefully considered when surface modifications are made to AuNPs. This is especially important for the delivery of drug molecules to ensure that the cargo is not lost before the intended destination. Electrostatic interactions might be desirable, since covalent bonds require energy for the cargo to dissociate from the surface. A similar dissociation of surface attachments that had electrostatic interactions with the nanoparticle surface has been reported for superparamagnetic iron [28,29].

The effect of the dose was more prominent for the Au compared to the citrate surface coating. With the exception of the liver, the %ID/g was higher in the lower dose level in all of the organs/tissues. For citrate, the opposite was observed: the %ID/g was lower in the higher dose level for blood and lung (a blood-rich organ). This can possibly be explained by isotopic exchange between the citrate (which predominates in blood) and its radiolabeled analogue. With higher dose, the amount of ^14^C citrate will be the same in the blood as that in the lower dose, thus giving a lower %ID/g. The ratios of the %ID/g of the 90 μg:9 μg dosages for Au (liver: 2.6; spleen: 0.6; lungs: 0.8; blood: 0.8) may be an indication of a saturable transport mechanism of the Au into tissues/organs, with the liver taking up excess Au in the case of higher dosing levels. If this can be repeatedly shown, it may be a useful consideration when planning to use AuNPs as a drug delivery vector. To date, there is little evidence that AuNPs lead to histological changes and toxicity [33,44]. Whether this will be the case in an extensive treatment regime, with multiple doses administered over the course of weeks or months remains unknown. It is also not known whether the systemic/tissue concentrations will be maintained by the prolonged exposure of the repeated doses, unlike in this acute study. The subchronic and chronic use of AuNPs presents another variable and so does the level of biopersistence. All of the above scenarios will need to be investigated.

## 4. Experimental Section

### 4.1. Preparation of AuNPs and Dual-Radiolabeled AuNPs

Elemental gold (24 carat) was purchased from Cape Precious Metals Holding Pvt. Ltd., Johannesburg, South Africa. 1,5-[^14^C] citric acid (concentration: 3.7 GBq/mL; specific activity: 2.07 GBq/mmol) was purchased from American Radiolabeled Chemicals, Inc. (St. Louis, MO, USA). Hydrochloric acid (HCl, 37%), nitric acid (HNO_3_, 68%) (used to prepare aqua regia using an HCl:HNO_3_ in a 3:1 ratio) and trisodium citrate (Na_3_C_6_O_7_H_5_·H_2_O), were all of analytical grade and purchased from Merck (Billerica, MA, USA). Deionized water (resistance >18 MΩ) was prepared by an in-house ultrapure water system (Merck Millipore, Billerica, MA, USA). All chemicals, except for the 1,5-[^14^C] citric acid (deprotonated using NaOH to make trisodium citrate), were used as received without purification. All radioactive materials were produced and handled at the South African Nuclear Energy Corporation (Necsa, Pelindaba, South Africa) facilities and laboratories.

Two 5-mg samples of natural gold (^197^Au) metal were weighed using an analytical balance (5-decimal place Mettler Toledo). One sample was used as natural gold, while the other sample (target) was irradiated in the SAFARI 1 20 MW research reactor situated at Necsa in a hydraulic position with a neutron flux of 0.5 × 10^14^ n∙cm^−2^∙s^−1^ for 20 min to obtain ^198^Au. Both Au samples were dissolved in aqua regia (5 mL) dried down (using heat) and reconstituted in 0.5–1 mL 0.005 N HCl to yield HAuCl_4_∙HAuCl_4_ and [^198^Au]HAuCl_4_∙[^198^Au]HAuCl_4_ in 0.05 N HCl [45], the starting material in the synthesis of AuNPs. The activity of the [^198^Au]HAuCl_4_∙[^198^Au]HAuCl_4_ was measured using a CRC-15R dose calibrator (Capintec Inc., Ramsey, NJ, USA). The radioactive HAuCl_4_ sample was used to synthesize the dual-radiolabeled AuNPs. The activity concentration of 1,5-[^14^C]trisodium citrate was determined by liquid scintillation. Three counting solutions were used to determine the activity concentrations of the 1,5-[^14^C]trisodium citrate. These solutions were prepared using a standard containing 10 μL (37KBq) of 1,5-[^14^C]trisodium citrate whose volume was made up to 1 mL (stock solution). Five, then and one hundred microliters of the stock solution were added to 20-mL glass vials containing 15 mL of the liquid scintillation cocktail (Bioscint). The activity measurements in the vials were 8491, 14,420 and 139,818 disintegrations per minute (DPM), respectively.

An adaptation of the method published by Turkevich, *et al.* [46] and Frens [47] was used to synthesize sterile radioactive and natural AuNPs. The volumes of the prepared solution of radioactive [^198^Au]HAuCl_4_∙[^198^Au]HAuCl_4_ in 0.05 N HCl and the non-radioactive HAuCl_4_∙HAuCl_4_ in 0.05 N HCl were diluted to 25 mL using deionized water to make 1 mM solutions. Solutions of hydrogen chloroauric acid were heated to the boiling point with vigorous stirring, and the reducing agents were added to the solutions and boiled under reflux for a further 30 min. For the non-radioactive synthesis, 2.5 mL of 38.8 mM trisodium citrate were used as the reducing agent. For the dual radiolabel synthesis, 2.5 mL (38.8 mM) of solution containing 1,5-[^14^C]trisodium citrate (1.52 MBq: 600 μL, 1.07 × 10^−3^ mmol) and non-labeled trisodium citrate (1.9 mL: 9.743 × 10^−2^ mM) were used as the reducing agent. Figure 4 shows the adapted method used to synthesize dual-radiolabeled [^14^C]citrate-[^198^Au]AuNPs.

### 4.2. Characterization of Dual-Radiolabeled AuNPs

Both the radioactive and non-radioactive AuNPs were characterized using the same techniques to assess the impact of using radioactive precursors in the quality attributes of AuNPs. With the exception of the UV/Vis spectra, the radioactive sample was analyzed after 10 half-lives (27 days), when the radioactivity of the samples was low enough to be safely cleared from Necsa laboratories and analyzed in non-radiological laboratories.

The hydrodynamic size (Z-average size) and polydispersity index (PDI) of the nanoparticles was acquired by dynamic light scattering with a Zetasizer Nano ZS (Malvern Instruments Ltd., Worcestershire, UK) operated in backscattering mode at 173° with a He–Ne laser beam (λ = 632.8 nm). For the zeta potential measurements, which were performed at 25 °C with a scattering angle of 90°, the particles were dispersed in aqueous solution with an average pH of 6.2. The experiment was done in triplicate, and the results were averaged.

The morphology and primary size distributions of AuNPs were determined using transmission electron microscopy (TEM) (FEI Tecnai G2, Eindhoven, The Netherlands). Specimens were prepared by drop casting of a 10-μL aliquot of a dilute NP solution on an Athene^®^ grid (Plano GmbH, Wetzlar, Germany). At least 250 particles were used to determine the primary size distributions using ImageJ software (Version 1.48; National Institutes of Health, Bethesda, MD, USA).

UV/Vis spectra were recorded for both the radioactive and non-radioactive AuNP suspensions using a PerkinElmer LAMBDA 1050 UV/Vis/NIR spectrophotometer (Waltham, MA, USA). The spectra were also used to determine the concentration [48].

### 4.3. In Vivo Study

#### 4.3.1. Animals

The study was conducted in accordance with the South African National Standard for the Care and Use of Animals for Scientific Purpose. Ethical approval was sought and granted by the North-West University (NWU) Ethical Committee. Twelve (12) male Sprague Dawley rats, 8–10 weeks old, weighing 200–250 g, were used in the study. The rats were bred and procured from the Department of Science and Technology (DST)/NWU/Preclinical Drug Development Platform (PCDDP) Vivarium (Potchefstroom, South Africa) and housed in stainless steel cages in groups of 4. The rats were kept under standard environmental conditions (23 ± 1 °C, 55% ± 5% humidity and 12/12 h light/dark cycle) with water and food provided *ad libitum* throughout the study.

#### 4.3.2. Experimental Design

**Figure 6:**
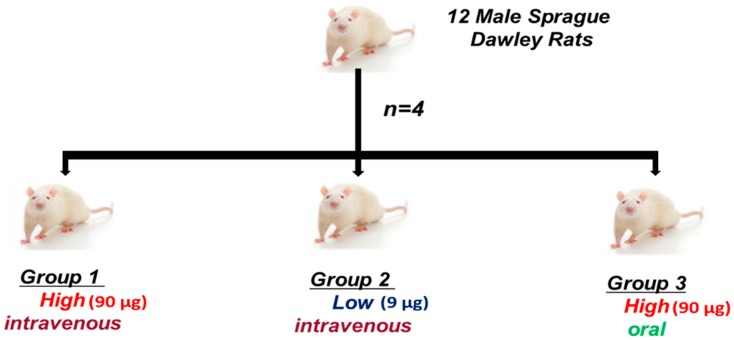
Study design of the animal experiment. Groups 1 and 2 received intravenous doses, while Group 3 got an oral dose.

The rats were randomly divided into 3 treatment groups (*n* = 4 per group; see Figure 6). The dual-radiolabeled [^14^C]citrate-[^198^Au]AuNPs suspensions were administered as a slow intravenous injection using the tail vein in Groups 1 and 2 and orally via gavage in Group 3. The administered doses were 90 μg (high dose) for Group 1 and 9 μg (low dose) for Group 2. The administered doses were within the ranges found in the literature [13]. The volume of all of the administrations was 500 μL. The accuracy of the dose was controlled using both the volume injected and the radioactivity of ^198^Au. Any activity remaining in the syringe was measured and used to calculate the exact dose injected.

After each administration, the rats were individually placed in metabolic cages to collect the total amount of urine and feces. All of the administrations were well tolerated with no apparent adverse events being observed during the 24-h study. The 24-h time point was selected based on acute biodistribution studies found in the literature [20,22]. At the termination of the study, the rats were euthanized using an overdose of Euthapent^®^ (sodium pentobarbitone 200 mg/mL; Kyron laboratories, Johannesburg, South Africa), administered intravenously. Blood was collected using the cardiac puncture technique and stored without further processing before cutting open the chest cavity and abdomen of the rat to collect the liver, spleen and lungs. Together with the blood, these were used to determine whether the gold core of the AuNPs is distributed in a similar pattern as the citrate surface coating, which is an indication of whether the NPs remain intact in physiological conditions. The mass of each sample, including the carcass, was measured and used in calculating the percentage of the injected dose per gram of the organ/tissue.

### 4.4. Quantification of Gold and Citrate in Samples

The quantification of citrate was done after at least 30 days (10 half-lives) of ^198^Au, to avoid measuring the *beta* decay of ^198^Au, as well. The quantities of citrate were determined only in the intravenous groups, since no absorption of Au was seen, thus negligible (≥0.001 %ID/g) amounts in the blood, liver, lungs and spleen (results not shown).

#### 4.4.1. Gold

The ^198^Au radioactivity of the blood, liver, lungs, spleen and the remainder (total remaining carcass) was measured without further sample preparation by γ-spectroscopy using a CRC-15R dose calibrator (Capintec Inc., Ramsey, NJ, USA) and a lead-shielded well-type NaI (TI) scintillation detector using the winTMCA32 software (FLIR Radiation, GmbH, Solingen, Germany). The counts were corrected for physical decay from the time of injection and any background radiation. A ^198^Au standard prepared in the laboratory was used to correlate ^198^Au radioactivities to the masses, numbers and surface areas of the AuNP suspension used in the study. To ensure that the entire administered dose was accounted for, the amounts of ^198^Au in the total urine and feces and the total remaining carcass was measured. A gamma spectrum of the ^198^Au was also measured to give evidence that the signal measured was only from gold, and there were no interferences that may impede the reliability of the results.

#### 4.4.2. Citrate

To measure the ^14^C radioactivity in liver, lungs, spleen and blood, a known mass of approximately 200 mg of the liver, lungs and spleen and 500 μL of whole blood were added to a 20-mL glass scintillation vial. To each sample, 1–2 mL of the solubilizer (Biosol, National Diagnostics, Atlanta, GA, USA) were added, and the samples were incubated between 55 and 60 °C until the samples were completely solubilized or had a brown/green color in the case of the blood. The digestion times varied depending on the tissues (up to 5 h for liver). Two hundred microliters of 30% hydrogen peroxide (H_2_O_2_) were added in 2 aliquots to discolor the dissolved tissues. The samples were allowed to stand for 24 h. Scintillation cocktail (Bioscint, National Diagnostics, Atlanta, GA, USA) was added to fill up the vial to 20 mL. The samples were stored in a cool dark place and counted for 10 min using a Perkin-Elmer Tri-Carb 3100 TR scintillation spectrophotometer (Waltham, MA, USA). All measurements were done in triplicate.

### 4.5. Statistics

The statistical significance of the differences between the mean %ID/g values in the different groups was assessed by use of the non-parametric Mann–Whitney test and a Student *t*-test. Statistical probability (*p*) values less than 0.05 were considered significantly different.

## 5. Conclusions

With the present study, we have shown that the use of radioactive precursors does not have a negative impact on the physico-chemical properties of AuNPs, and dual radiolabeling is a good technique for studying the biodistribution of a multi-component nano-particulate system. The biodistribution profile of the Au core and citrate surface coating are different, and for the Au component, the biodistribution is dose dependent. At both dose levels, the majority of the Au accumulates in the liver and spleen, and an unexpected deposition in the lungs occurs after intravenous administration.
